# Activation of the 26S Proteasome to Reduce Proteotoxic Stress and Improve the Efficacy of PROTACs

**DOI:** 10.1021/acsptsci.4c00408

**Published:** 2024-12-16

**Authors:** Jindrich Sedlacek

**Affiliations:** † Department of Genetics and Microbiology, Charles University and Research Center BIOCEV, Pru°myslová 595, Vestec 252 50, Czech Republic; ‡ Institute of Organic Chemistry and Biochemistry of the Czech Academy of Sciences, Flemingovo náměstí 2, 16610 Prague, Czech Republic

**Keywords:** Proteasome, PROTAC, cAMP, cGMP, p38 MAPK, NFE2L1, NFE2L2, USP14

## Abstract

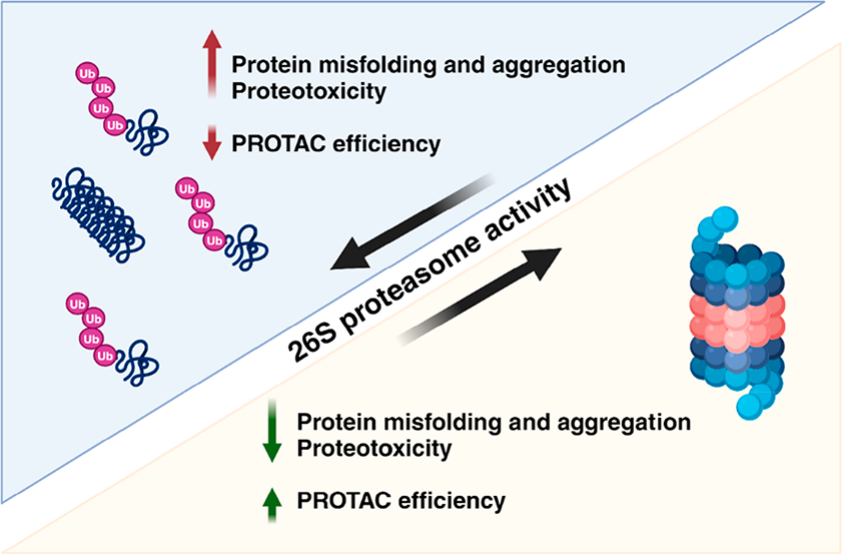

The 26S proteasome degrades the majority of cellular proteins and affects all aspects of cellular life. Therefore, the 26S proteasome abundance, proper assembly, and activity in different life contexts need to be precisely controlled. Impaired proteasome activity is considered a causative factor in several serious disorders. Recent advances in proteasome biology have revealed that the proteasome can be activated by different factors or small molecules. Thus, activated ubiquitin-dependent proteasome degradation has effects such as extending the lifespan in different models, preventing the accumulation of protein aggregates, and reducing their negative impact on cells. Increased 26S proteasome-mediated degradation reduces proteotoxic stress and can potentially improve the efficacy of engineered degraders, such as PROTACs, particularly in situations characterized by proteasome malfunction. Here, emerging ideas and recent insights into the pharmacological activation of the proteasome at the transcriptional and posttranslational levels are summarized.

The maintenance and dynamic regulation of the healthy and fully functional proteome requires precise control of protein synthesis, folding, trafficking, and degradation. The degradation of proteins in the cell is a continual process that is precisely regulated and ruled by two major proteolysis mechanisms, the proteasome-mediated pathway and the lysosomal degradation pathway.^1−3^ The major degradation mechanism in eukaryotic cells is the proteasome pathway, which is responsible for the breakdown of approximately 80% of all cellular proteins, most of which are labeled with ubiquitin (Ub).^4^ Protein degradation is regulated through the ubiquitin proteasome system (UPS). This tightly controlled degradation system plays a crucial role in controlling all cellular functions, such as the cell cycle and signaling, the regulation of gene expression, inflammation, cell differentiation, and apoptosis. Its importance, especially in the case of abnormal protein expression, accumulation and/or activity, is associated with diseases, especially cancer and neurodegeneration.^2,4−6^ In general, the rate of protein degradation can also differ in response to physiological stimuli, development and aging, and disease. Modulation of the turnover rate of different proteins is relevant for therapeutic use because it has a broad impact on physiological processes in different cells and tissues.^7−9^

Ubiquitin-mediated proteolysis is the process by which ubiquitin, a 76-amino acid residue-containing protein, is covalently bound to the targeted protein mainly in the form of polyubiquitin chains and is subsequently labeled for degradation via the 26S proteasome. In the cell, chains that are connected via one of the seven lysine residues (K6, K11, K27, K29, K33, K48, and K63) of ubiquitin and through N-terminal methionine-linked ubiquitin (Met1-Ub) chains exist.

The rates of protein degradation via the UPS pathway are generally assumed to be determined by chain length and the rate of K48 ubiquitination.^10−12^ Mono- or polyubiquitination also regulates the function of many proteins under various physiological or pathological conditions.^11,12^ However, four different chain topologies (Lys6, Lys27, Lys29, and Lys33) and monoubiquitylation result in different outcomes in the cell, indicating that ubiquitylation serves as a code to transmit and store information. Currently, their significance is only poorly understood.^10,12^

The ubiquitination of proteins requires three enzymes. In the initial step, the ubiquitin-activating enzyme (E1) catalyzes ubiquitination reactions. In this first and crucial step, E1 binds to the C-terminal glycine residue of ubiquitin in an ATP-dependent manner. The binding of Ub to a cysteine residue on E1 occurs via a thioester bond to the form of the Ub-E1 complex. In the next step, the ubiquitin-conjugating enzymes (E2s) transfer ubiquitin from the Ub-E1 complex to an E2 cysteine by trans-thioesterification to form the Ub-E2s complex. Finally, E3 ubiquitin ligases transfer and covalently bind Ub from the E2 cysteine to a lysine residue on the target protein. Hence, E3s are tightly regulated to ensure accurate substrate ubiquitylation. Mammals have two E1s, approximately 40 E2s, and are estimated to have more than 800 E3s.^13,14^ The conjugation of Ub to substrates is reversible, and the reverse process, known as deubiquitination, is catalyzed by deubiquitinases (DUBs), also known as deubiquitinating isopeptidases, which can remove ubiquitin from target proteins, thereby reversing the effects of ubiquitination. This process also allows the recycling of ubiquitin for future use.^4,5,12^ For instance, the acetylation of lysine residues can also inhibit the formation of Ub chains.^14^

The 26S proteasome is a 2.5 MDa multicatalytic protein complex composed of a 20S core particle and one or two ATP-dependent 19S regulatory particles attached to the end(s) of the 20S particle.^15,16^ The proteasome is also referred to as the 26S/30S proteasome when the 20S is capped at one or each end with the 19S regulatory particle. Only capped complexes (predominantly 26S) are responsible for ubiquitin-dependent proteasome degradation. The 20S core particle is a threonine-based protease that is composed of four stacked rings. The two inner β-rings composed of the β1−β7 subunit contain three catalytically active subunits (β5, β2, and β1) that exhibit chymotrypsin-like, trypsin-like, and caspase-like activity, respectively. The two exterior heptameric α-rings (α1−α7) serve as gated channels that regulate substrate translocation and product exit from the inner catalytic chambers.^17−21^ These outer rings also act as docking surfaces for the 19S subunit, also known as PA700, which binds to the 20S subunit and promotes gate opening of the 20S subunit for proteolytic degradation of polyubiquitinated protein substrates.^17^ The 19S subunit is comprised of six AAA+ ATPase subunits (Rpt1–Rpt6) and 13 non-ATPase subunits (Rpn1-Rpn3, Rpn5–Rpn13, and Rpn15). The ATPase subunits form a heterohexameric structure that mediates substrate recognition, unfolding, 20S particle gate opening, and substrate entry into the 20S catalytic particle.^22−24^ Rpn1, Rpn10, and Rpn13 bind to ubiquitin chains through a unique motive for recognizing ubiquitin or ubiquitin-like domains.^22−24^ Therefore, 19S provides an unusually versatile binding platform that can identify substrates for degradation by polyubiquitin chains that differ greatly in length and topology. Two other proteasome activator families (called 11S), PA28/REG/PA26 and PA200/Blm10, are also known to bind and activate the 20S core proteasome for protein degradation.^25^ In addition, ZFAND5 is a factor that also interacts with the 26S proteasome and increases its ability to hydrolyze ubiquitin-labeled substrates.^26^ These regulators are less conserved than ATP-dependent activators, and their roles are less defined, although the molecular mechanisms through which they activate proteasomes have been revealed.^25−27^ Many advances in understanding regulatory processes have been obtained recently through many studies and by cryogenic electron microscopy of the 26S complex. However, this mechanism is a multistep process, and many key features of how the proteasome works are not yet fully understood.^4,28−30^ The proteasome’s degradative capacity is not fixed, and the proteasome is not simply an apparatus for the destruction of Ub-labeled proteins and for ubiquitin recycling (a strict mechanistic view of life); rather, it is tightly regulated by multiple postsynthetic mechanisms and the work of other enzymes in the UPS cascade.^4,25,27^ In contrast, the 20S catalytic proteasome particles are ATP and proteasome activator-independent complexes that degrade diverse substrates. Notably, these substrates are strongly enriched in nucleic acid-binding proteins with intrinsically disordered domains commonly found in nuclei and nuclear stress granules.

Elevated 20S proteasome levels also contribute to cell survival during stress conditions associated with damaged protein forms.^31−33^ The most prevalent neurodegenerative diseases (e.g., Alzheimer’s, Huntington’s, and Parkinson’s diseases) are characterized by the accumulation of aggregation-prone protein species with effects on the loss of proteasome function, which leads to progressive neuronal cell death. The accumulation of aggregation-prone proteins, such as β-amyloid, α-synuclein, and polyglutamine repeats, which are undruggable to conventional low-molecular-weight drugs, is a typical hallmark of neurodegeneration. Some therapeutic strategies for treating these disasters attempt to restore affected proteins back to their natural state or suppress their accumulation.^6^ Current techniques mostly target receptor or enzymatic proteins, which can be modulated with specific small molecules.^34^ However, this classic paradigm is limited by the number of druggable proteins; approximately 10% of the human proteome represents druggable targets (e.g., nuclear hormone receptors, G-protein coupled receptors, ion channels, protein kinases, and diverse proteases), and only half of those are relevant to disease. Owing to the lack of active sites (e.g., scaffolding and nonenzymatic proteins or transcription factors), a large part of the human proteome is undruggable via this strategy; therefore, efforts to broaden usable proteome targets have been a recent focus.^5,34,35^

An idea exploited for this purpose is the recruitment of a cellular degradation cascade (UPS) for targeted protein removal using proteolysis-targeting chimeras (PROTACs). Additionally, PROTACs can rapidly and specifically degrade proteins of interest (POIs) at the posttranslational level by coopting with the ubiquitin–proteasome system. Even so, PROTACs are still an emerging technology, and their future development is fraught with many challenges.^35^ Nevertheless, the phenomenon of proteasome malfunction can also strongly affect the degradation of POI mediated by PROTAC molecules. Low-molecular-weight compounds that increase 26S proteasome activity can effectively increase the clearance of toxic protein species, which are strongly connected with proteasome malfunction and can increase PROTAC-mediated degradation of POIs in the case of proteasome insufficiency.^6,36^ Therefore, in this Review, strategies for increasing ubiquitin-dependent 26S proteasome degradation flux are addressed. It can play an important role in novel therapies for protein conformation diseases or can be used to improve the effect of engineered degraders for unique targets in cases of proteasome impairment.

## 1 Targeted Protein Degradation

### 1.1 Proteolysis-Targeting Chimeras (PROTACs)

**Figure 1:**
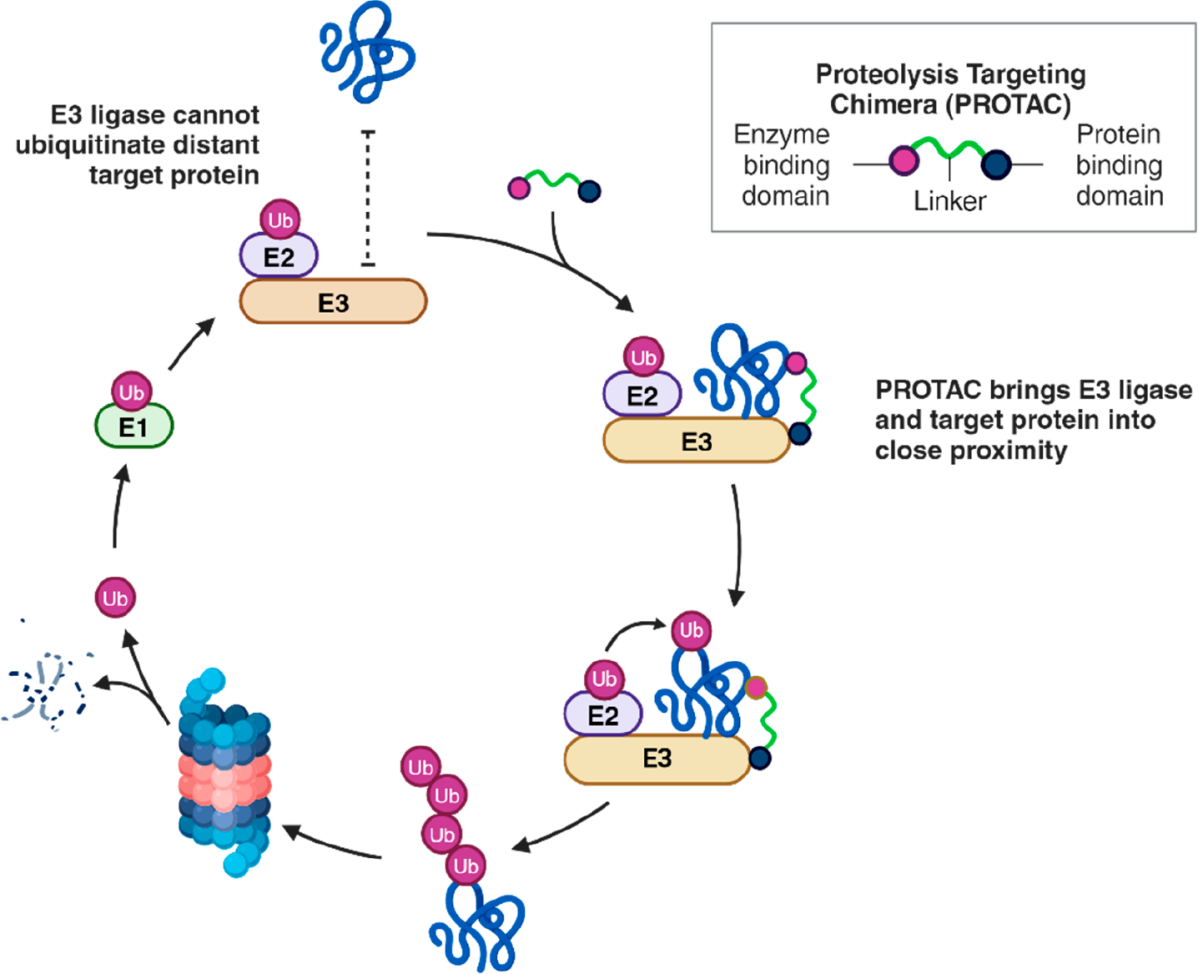
Schematic representation of PROTAC technology. Protein of interest (POI) degradation via the ubiquitin–proteasome system mediated by PROTAC. A PROTAC molecule consists of three different parts: an E3 ligase-recruiting ligand, a ligand that binds to the POI, and a linker that connects these two ligands. A single PROTAC molecule can promote the breakdown of many protein molecules.

PROTACs are a novel paradigm-changing technology that uses the ubiquitin–proteasome system for targeted protein degradation (Figure 1). PROTACs form ternary complexes linking E3 ligases or their substrate receptors (e.g., pVHL, βTrCP1, CRBN, Mdm2, DCAF15, DCAF16, RNF114, and c-IAP1) and substrates, which are typically disease-related proteins.^37,38^ The largest superfamily of E3s are Cullin-RING ligases (CRLs), with over 300 known members in humans. CRLs are also one of the most important cellular regulatory systems, and their substrates are exclusively defined by the identity of a CRL’s substrate receptors, which are extensively employed by PROTACs.^14,37−39^

This chemical induction of close proximity between the E3 ligase and POI causes polyubiquitination and subsequent 26S proteasome-mediated degradation.^37^ The event-driven mechanism of PROTAC action provides several advantages over traditional occupancy-driven small-molecule inhibitors, including their catalytic nature, reduced dosing and frequency, more potent and persistent effect, increased selectivity to reduce possible harmful toxicity and efficacy in the face of common drug resistance mechanisms, ability to target nonenzymatic functions and extend the target space.^37,38,40^

Therefore, PROTACs have demonstrated significant therapeutic potential by degrading multiple disease-related and causative proteins.^40^ For instance, PROTACs allow the therapeutic exploitation of proteins that were considered undruggable (e.g., bromo- and extraterminal domain (BET) family proteins and transcription factors such as c-MYC). In addition to interfering with ligand interaction sites, PROTACs activate protein breakdown, which also enables the inhibition of protein scaffolding functions or previously undruggable protein–protein interactions.^35,37,38,40^ These heterobifunctional molecules are composed of two active domains joined by a linker that lacks ideal properties; moreover, these heterobifunctional molecules still need refinement.^37^ In the past 20 years, since the concept of PROTAC molecule employing the ubiquitin–proteasome system to degrade a target protein was reported, PROTACs have gone from academia to the pharmaceutical industry, where many innovative companies have announced projects in preclinical and early clinical stages of development.^40,41^ The first two PROTAC drugs, ARV-471 targets the estrogen receptor in metastatic breast cancer, and ARV-110 targets the androgen receptor in metastatic castration-resistant prostate cancer, has entered phase II clinical trials. These two PROTACs has shown strong clinical performance in phase I clinical trials. If phase II and III clinical trials will be successful, they could pave the way for the widespread clinical use of PROTACs.^41^

### 1.2 PROTAC Limitations

Traditional PROTACs are chimeric compounds that bind to a target protein in the cell, and via dual bonding, mediate its proximity with the E3 ubiquitin ligase.^40^ Progress in organic synthesis, medicinal chemistry, and molecular pathology has allowed the creation of different conjugates and the assessment of their physicochemical and biological properties, first in cell cultures and animal models. However, PROTACs have several drawbacks.^40,41^ First, PROTACs can induce nontargeted degradation of various other proteins.^42^ For example, PROTACs containing the immunomodulatory drugs (IMiDs) thalidomide, pomalidomide, and lenalidomide, which function as ligands for the CRBN E3-ubiquitin ligase, degrade not only the desired target but also other proteins, such as the transcription factors SALL4 and Ikaros (IKZF1 and IKZF3) or the translation regulator GSPT1.^42^ Moreover, PROTACs are high-molecular-weight compounds, and their physical and chemical properties fall outside Lipinski’s rule of five.^43^ Therefore, the molecular properties of PROTACs, which are important for the optimization of their structures, need to be addressed. Most published PROTACs has been evaluated only at the molecular or cellular level, while data on their pharmacokinetics and pharmacodynamics are scarce.^44^ However, an extensive reanalysis of the physicochemical properties of 1806 PROTAC compounds administered orally or intravenously to rats was performed to better describe and understand the determinants of oral availability to optimize the physiochemical characteristics of diverse parts of PROTAC molecules for better peroral absorption.^45^ In addition, several examples of optimized absorption, distribution, metabolism, and excretion (ADME) profiles exist. Thus, there is insufficient information to monitor ADME characteristics to optimize their potential as oral drugs.^43−45^ Heterobifunctional PROTACs are flexible and conformationally complex compounds. Therefore, simple descriptions of their molecular properties (size, shape, polarity, lipophilicity, and degree of ionization), which are utilized to characterize small-molecule inhibitor properties, cannot be simply applied to the chemical space of PROTACs to discover candidates.^46^ Solubility and permeability are key parameters for determining the bioavailability of PROTACs. Common tests, such as artificial membrane permeability and the Caco-2 cell permeability assay, are not fully relevant for evaluating the distribution of PROTACs because of concerns about their solubility in assay buffers and nonspecific binding. These tests should be modified to better characterize the complex properties of PROTACs to improve their pharmacokinetics and pharmacodynamics with the possible impact on their oral availability.^38,47^ Solubility has a crucial role in the success of PROTACs. The poor solubility and insufficient understanding of this phenomenon limit various phases of the drug discovery process. In addition, the connection between solubility and permeability makes their simultaneous optimization a difficult challenge for medicinal chemists. Increasing permeability through increasing lipophilicity usually decreases the solubility and metabolic stability of PROTAC molecules. It is generally believed that an acceptable solubility is a premise for achieving sufficiently high blood concentrations to obtain a significant therapeutic effect *in vivo*.^45,48^

In addition, PROTACs are not required to completely occupy the target binding site for the duration of their action. Indeed, the catalytic action of PROTACs involves the formation of stable ternary complexes (E3-PROTAC-POI) that can compensate lower solubility and membrane permeability, as has been shown for the MZ1 degrader of BET family proteins (BRD4, BRD3, and BRD2).^49,50^ However, identification of optimal ternary complexes is often challenging without a trial-and-error approach, which requires the synthesis and testing of many different PROTAC variants.^51,52^

Notably, among the E3 enzyme ligands most widely used in the design of PROTACs, only IMiDs (thalidomide derivative molecules) are suitable for oral bioavailability. Hot clinical candidates such as ARV-110 and ARV-471 contain CRBN ligands, whereas pVHL, c-IAP1, and MDM2 ligands have higher molecular weights, larger topological polar surface, and greater flexibility and are thus potentially unfavorable for oral use.^53,54^ On the other hand, CRBN ligands have low chemical and metabolic stability because of racemization of the glutarimide group and spontaneous hydrolysis of imides. This problem can be solved by developing a new E3-ligand to improve bioactivity through increased membrane permeability or pharmacologically aimed efficiency, decreasing the half-degrading concentration (DC50) of PROTAC-mediated protein degradation.^54^

Furthermore, many diseases are associated with significant decreases in proteasome catalytic activity.^6^ In such cases, even if a PROTAC can ubiquitinate a POI, degradation may not be effective. Pharmacological strategies such as an increase of 26S proteasome flux to improve the efficiency of PROTAC-mediated degradation can also expand the effective concentration range to lower effective cellular concentrations to avoid the concentration-dependent hook effect caused by the accumulation of undegraded binary (E3-PROTAC, POI-PROTAC) complexes at excessively high concentrations of the PROTAC molecules. This effect negatively impacts the apparent potency of PROTACs in a concentration-dependent manner.^40,42,55^

Importantly, PROTACs cannot solve the issue with proteasome malfunction and instead are limited by this restriction since they rely on healthy endogenous proteasomes to degrade target proteins.^4,6^ Consequently, for PROTACs to be effective at treating certain disorders, they may have to be combined with pharmacological strategies to increase proteasome function. Therefore, factors that can positively affect 26S proteasome Ub-dependent degradation and offer pharmacological solutions for improving the effectiveness of PROTACs in systems with proteasome-mediated proteostasis deficits, such as neurodegenerative or cardiovascular diseases, are discussed.^4,6,56−59^

## 2 Mechanisms of Enhancing 26S Proteasome Activity

### 2.1 An Increase in Proteasome Content through Transcriptional Regulation

**Figure 2:**
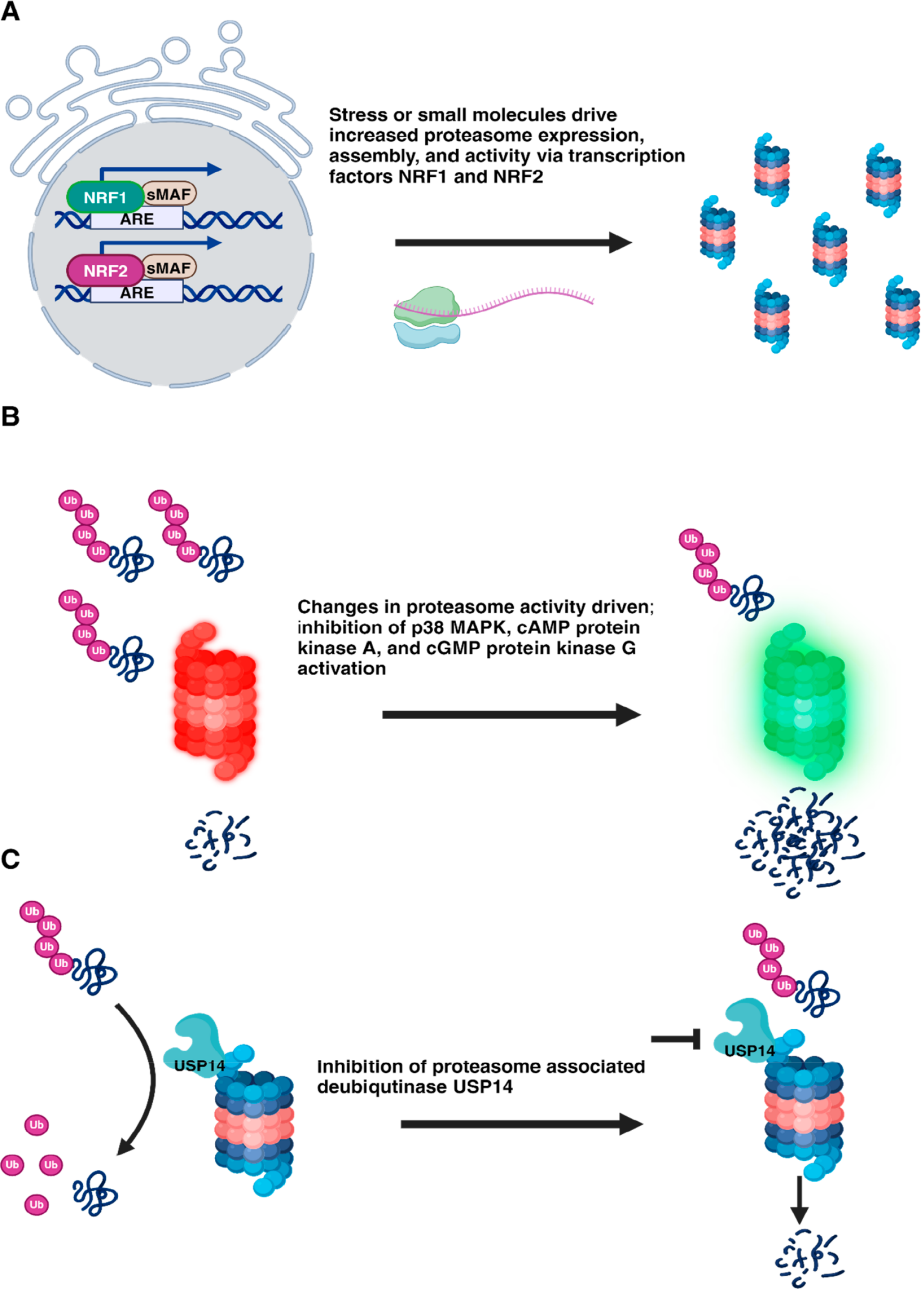
Increasing 26S proteasome activity. (A) Genetic upregulation of proteasome activity via the transcription factors NRF1 and NRF2. (B) Enhancing proteasome degradation by the modulation of posttranslational modifications; inhibition of p38 MAPK signaling and cAMP protein kinase A and cGMP protein kinase G activation, resulting in enhanced 26S proteasome-mediated proteolysis. (C)USP14 inhibition can promote 26S proteasome-mediated protein degradation by preventing ubiquitin chain trimming.

The 26S proteasome is comprised of 33 distinct subunits, and its biogenesis is tightly regulated at several steps, such as transcription, subunits folding, particle assembly, and posttranslational modifications.^4,60,61^ The abundance of proteasome subunits in the cell is controlled by different transcription factors (e.g., NRF1/NRF2 or FOXO1/FOXO4).^60−62^ Among these transcription factors, two more significant are the cap-’n’-collar basic leucine zipper (CNC-bZIP) transcription factors NRF1 and NRF2 (also known as NFE2L1 and NFE2L2 in mammals). The ChIP and RNA-seq data sets revealed that the target genes of NRF1 and NRF2 overlay slightly, however NRF1, not NRF2, mostly regulates the expression of all proteasome components and genes related to proteostasis in general.^62−64^ NRF1 knockout or knockdown suppresses the expression of proteasome genes and *de novo* proteasome formation in response to proteasome insufficiency (Figure 2, A); moreover, recent studies revealed that NRF1 is a key player that coordinately upregulates the expression of all proteasome subunit genes, prevents proteasome malfunction and increases proteasome activity.^60,65−67^ The second key player, NRF2, is a short-lived transcription factor related to the expression a battery of cytoprotective genes involved in xenobiotic metabolism and the antioxidant response. A major emerging function of NRF2 is its role in resistance to oxidative stress. NRF2 can also regulate the synthesis of lysosomal and anti-inflammatory proteins and some 26S proteasome particle components.^64,68,69^ These properties, namely, protective action against oxidative stress, support a protective role of NRF2 against neurodegeneration.^68^ However, NRF2 does not induce expression of proteasome genes in a context-specific manner, unlike NRF1.^62−64^

Significantly reduced basal proteasome expression and decreased activity accompanied by increased accumulation of ubiquitinated proteins has been observed in the context of tissue-specific knockout of NRF1 in the liver and nervous system.^70−72^ NRF1 levels are strongly reduced in dopaminergic neurons in the substantia nigra of Parkinson’s disease patients.^73^ Decreased expression of the transcription factor NRF1 and its proteolytic activator DDI2 results in reduced proteasome activity in a mouse model of Kennedy’s disease.^74^ NRF1 plays a critical role in mammalian development, as demonstrated by the fact that knocking out NRF1 in mice is embryonic lethal.^75,76^ Additionally, tissue-specific depletion of NRF1, such as in the mouse brain and osteoblasts, results in neurodegeneration phenotypes and disrupted bone formation and size, respectively.^75−77^ These findings suggest that NRF1 has an essential and indispensable function in the basal regulation of proteasome genes in a context-dependent manner.^77^

Under physiological conditions, NRF1 is anchored into the endoplasmic reticulum (ER). The N-terminal transmembrane domain is inserted within the ER membrane, and the C-terminus of NRF1 is extruded into the ER lumen, where NRF1 is N-linked glycosylated. The NRF1 glycoprotein is continuously removed from the ER and ubiquitinated by the ER-resident ERAD factor HRD1.^60^ The process of extracting NRF1 from the ER requires the (VCP)/p97 ATPase complex. In cells with sufficient proteasome capacity, cytosolic NRF1 is rapidly degraded.^60,77,78^ In cells with insufficient proteasome activity, NRF1 degradation may decrease to the point at which NRF1 is proteolytically processed by the protease DDI2 and multyply deglycosylated by the peptide N-glycanase 1 (NGLY1), which removes N-linked glycans from asparagine residues and, via this mechanism, converts multiple asparagine residues in the protein sequence into aspartic acid. Only sequence-edited NRF1 promotes the expression of proteasome subunits and other protective genes by binding to antioxidant response elements (AREs) within their promoters. Elevated expression of all-proteasome subunit genes causes increased proteasome biogenesis, thus restoring and increasing proteasome mediated degradation.^60,77,78^ Additionally, several components of the autophagy–lysosomal pathway are upregulated in an NRF1-dependent manner, thus providing cells with an alternative way to overcome proteasome mall-function. In response to proteasome inhibitors, NRF1- or NGLY1-deficient cells exhibit several defects in the activation of autophagy and aggresome clearance.^79,80^

Studies on NRF1 activation have focused primarily on the response to proteasome inhibitors in hemato-oncology, and the detailed molecular mechanism underlying the nuclear translocation and activation of NRF1 is still not fully understood.^66,67,78^ A recent study in the roundworm (*Caenorhabditis elegans*) revealed that the switch on proteasome subunits expression by the orthologous gene SKN-1A is triggered by misfolded endogenous proteins as well as by expression of the human amyloid beta peptide, even though proteasome function is not harmed, indicating that SKN-1A activation is a widely protective mechanism against impaired proteostasis.^78^ In addition, activation of NRF1 and NRF2 by the small-molecule stimulator ASC-JM17 (Table 1, compound **1**) increases the proteasome content and related activity. This compound protects cells from the oxidative and proteotoxic stress induced by polyQ repeats and is active in fly and mouse models of Kennedy’s disease. ASC-JM17 also upregulates the expression of PSME1, a component of the proteasome 11S regulator, which is a part of the immunoproteasome, suggesting that this compound can induce structural rearrangement of proteasome complexes.^81^ Treatment-dependent induction of proteasome subunits was reduced by siRNA-mediated knockdown of NRF1 but not via NRF2. These findings show that ASC-JM17 acts through NRF1.^81^ Additionally, this substance has been granted FDA orphan drug status (UNII: 5VLL140BN9) for the treatment of spinocerebellar ataxia, Huntington’s disease, and Kennedy’s disease.

**Table 1:**
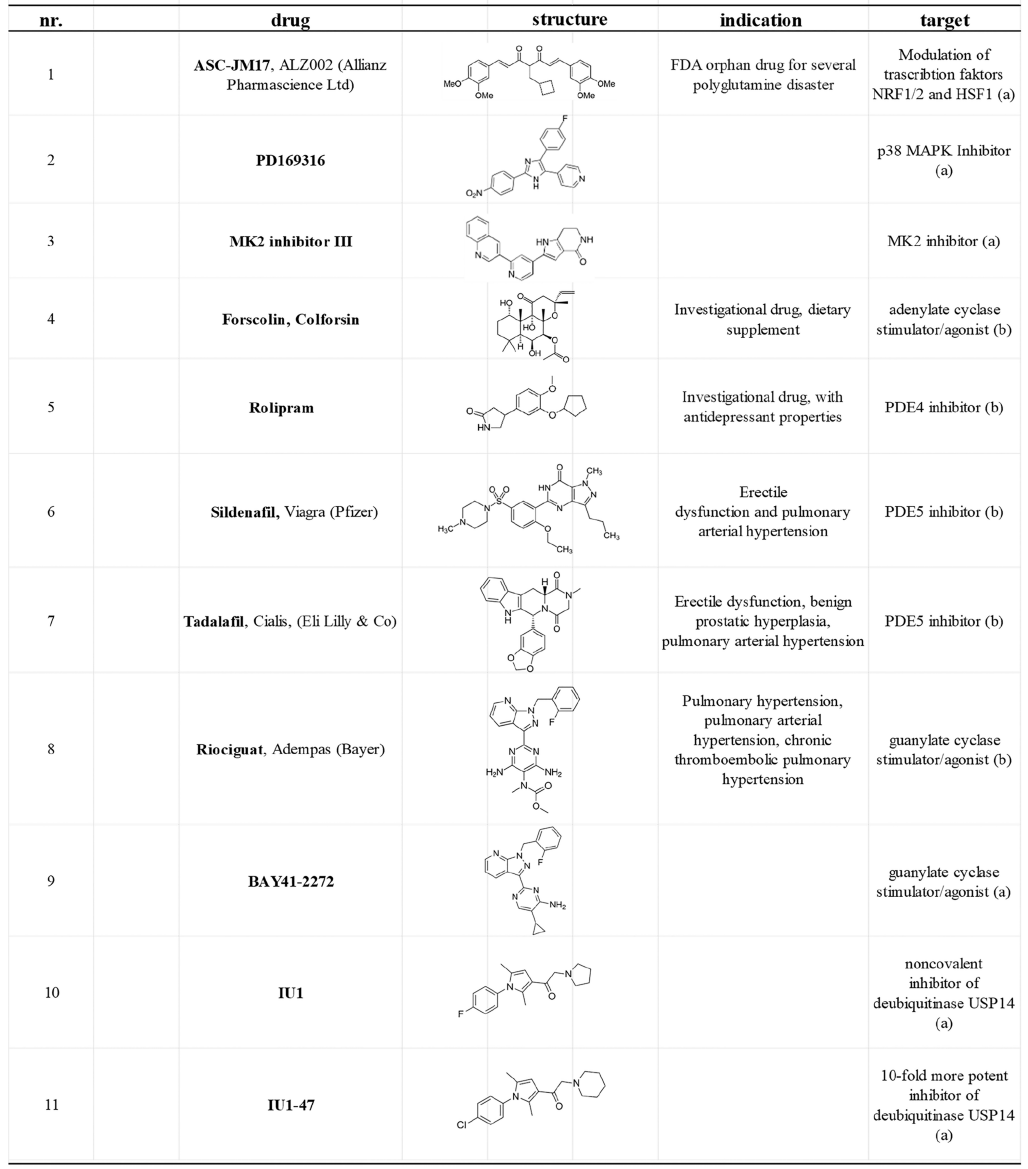
Compounds That Increase the Degradation Capacity of the 26S Proteasome^a^

a The information on indication and molecular targets is extracted from ChemBank/PubChem (a) or Drug Bank (b) unless otherwise annotated.

### 2.2 Proteasome Activation by Altering Posttranslational Modifications

#### 2.2.1 Increasing Proteasome Activity by Inhibiting p38 Mitogen-Activated Protein Kinase

p38 mitogen-activated protein kinase (p38-MAPK) is usually triggered by different proinflammatory and stress-inducing stimuli. Growing evidence suggests that this signaling pathway is involved in various biological responses other than inflammation, such as cell differentiation, proliferation, apoptosis, and invasion. It was suggested that p38-MAPK could be a potential therapeutic target for treating diseases such as chronic inflammation and cancer.^82,83^ Induction of the p38-MAPK pathway in cell lines and animal models causes tau phosphorylation, neuroinflammation, synaptic dysfunction and neurotoxicity, which are events linked with neurodegenerative diseases characterized by disruption of proteostasis, including amyotrophic lateral sclerosis and Alzheimer’s disease.^84−89^ Therefore, the search for p38-MAPK inhibitors has led to the development of approaches for targeting neurodegenerative diseases.^84^ Moreover, p38-MAPK is involved in proteasome phosphorylation, but this MAPK-dependent phosphorylation reduces proteasome activity in response to osmotic stress.^89^ Extensive knowledge of MAPK biological functions has been obtained due to the availability of specific inhibitors for several components of the MAPK signaling cascade.^82,83^

The genetic and chemical inhibition of either p38 MAPK and its upstream regulators, MAP kinase kinase 6 (MKK6) and apoptosis signal-regulating kinase 1 (ASK1), or its downstream target, such as mitogen-activated protein kinase (MAPK)-activated protein kinase 2 (MK2 or MAPKAPK-2), enhances proteasome activity.^36,83^ The most potent compound that increases proteasome activity via p38 MAPK inhibition is PD169316.^36^ p38-MAPK inhibition increases the activity of both the β1/5 and β2 subunits without affecting subunit abundance, cell survival, or the total amount of ubiquitinated proteins. The effect was confirmed in a panel of human cell lines.^36^ In contrast to treatment with PD169316 or the MK2 inhibitor III (Table 1, compounds **2** and **3**) alone, which only slightly activated cellular proteasomes, the combination of both compounds enhanced proteasome activity up to 4-fold in cell culture.^36^ These findings suggest that PD169316 and MK2 inhibitor III work synergistically to increase proteasome activity. p38-MAPK inhibition-mediated activation of 26S proteasome flux increases PROTAC-induced ubiquitin-dependent degradation of bromodomain-containing protein 4 (BDR4) without affecting overall protein turnover in cell cultures. These findings indicate that p38-related signaling inhibition increases ubiquitin-dependent protein degradation.^36^ In addition, the overall survival of cells expressing the toxic α-synuclein form is increased under treatment with p38-MAPK inhibitors.^36^ These findings highlight the potential of activating the 26S proteasome through p38-MAPK signaling. This effect can be accomplished through various mechanisms, including the employ of various small-molecule inhibitors.^36^

#### 2.2.2 Increasing Proteasome Activity by Activating cAMP-Dependent Protein Kinase A (PKA)

Briefly, cyclic adenosine monophosphate (cAMP) is produced by two families of adenylate cyclases (ACs), both of which belong to the class III AC superfamily. One family includes nine transmembrane ACs, AC1–9, which are activated by G-protein coupled receptors (GPCRs). These transmembrane ACs stimulate cAMP signaling in response to various extracellular stimuli. The second family includes soluble AC (AC10), which is distributed throughout the cytoplasm and within cellular organelles.^90,91^

cAMP acts as a second messenger in all types of cells, and this signaling pathway is involved in a variety of cellular life events. The well-characterized protein kinase A (PKA), whose activity is dependent on the cellular level of cAMP, phosphorylates the 19S subunit Rpn6, thus stimulating ubiquitin-dependent proteasomal degradation.^90^ In general, cAMP-dependent PKA activation enhances the capacity of cells to eliminate short-lived ubiquitinated proteins, including some aggregation-prone proteins (Figure 2, B). These conclusions are based on a wide range of biochemical and pharmacological findings.^90,91^

26S proteasomes are more active upon treatment with rolipram, a selective inhibitor of phosphodiesterase-4 (PDE4), which hydrolyses and inactivates cAMP. Also forskolin, which activates adenylate cyclase and is widely used as a standard method to increase cAMP levels, has a similar effect on the activation of 26S proteasomes in several cell lines and multiply stimulates proteasome activity in the brain upon the treatment of mice and zebrafish with rolipram (Table 1, compounds **4** and **5**). The administration of rolipram also led to decreased levels of aggregated tau *in vivo* and improvements in cognitive functions in mice.^92−95^ In contrast, these stimulatory effects on the proteasome can be blocked by the PKA inhibitor H-89.^92,96^ Additionally, calcium/calmodulin-dependent protein kinase II (CaMKII) promotes 26S proteasome activity by phosphorylating the Rpt6 subunit of the 19S proteasome subcomplex in the context of memory formation at the synapse.^97,98^ These findings show that both protein kinases phosphorylate Rpt6 to regulate proteasome function. However, PKA is likely more important for increasing proteasome flux, and its physiological relevance remains to be established *in vivo*.^96,99−104^

#### 2.2.3 Hormones Increasing cAMP Levels to Promote Proteasome Activity

cAMP and PKA signaling activation can also be mediated by diverse hormones and physiological conditions. The 26S proteasome can be activated *in vivo* by the physiological stimuli that increase cAMP levels in cell cytoplasm. The first cAMP-mediated metabolic response involves the stimulation of hepatic glycogen breakdown by epinephrine and glucagon.^93,94^ Treatment of murine hepatocytes with glucagon or epinephrine stimulates Rpn6 phosphorylation and increases the capacity of the 26S proteasome to degrade ubiquitinated proteins via activation of adenylate cyclase through a mechanism of action similar to that of forskolin. These hormones promote the breakdown of short-lived proteins, which include misfolded and some regulatory proteins but not the majority of other cellular proteins, in hepatocytes.^93,94^ In mouse kidney cells but not in cells with diminished PKA, treatment with antidiuretic hormone (ADH) stimulates proteasomal activity within 5 min, Rpn6 phosphorylation, and the selective breakdown of short-lived proteins. In the liver and skeletal muscles of mice starved overnight, cAMP levels, Rpn6 phosphorylation, and proteasome activity increase before any upregulation of autophagy and without any change in the level of the cellular proteasome.^92,94^ These observations in diverse cells and tissues clearly demonstrate that proteasome activation and enhanced degradation of short-lived proteins are physiological responses to cAMP and PKA action, not just pharmacological artifacts.^94,96^

Proteasomal activity and the capacity to degrade short-lived proteins seem to increase together with increased breakdown of triglycerides and glycogenolysis, which are induced during fasting in the liver, muscle, and heart tissue by epinephrine via cAMP.^96,105,106^ Nevertheless, depending on the study design and tissue, proteasome activity has been contradictory reported to increase rapidly upon nutrient deprivation, decrease slowly as a result of increased autophagy, or remain unchanged.^107−109^ Regardless of these contradicting mechanisms in cultured cells, the physiological mechanism involved in increasing the ability to protein degradation during fasting *in vivo* is through postsynthetic modification of cellular proteasomes, not via synthesis of proteasome particles.^94^

Enhanced proteasome activity in murine liver and muscle tissues is evident after 12 h of starvation and reflects a fairly quick metabolic reaction to food deprivation. This rapid response precedes the PI3K/Akt/FOXO axis-mediated induction of ubiquitin ligases and genes involved in autophagy, which causes muscle atrophy, especially the breakdown of myofibrils in muscles, which is observable after 1–2 days of food absence.^92,110−112^ Moreover, the FOXOs transcription factor-mediated response promotes the breakdown of long-lived proteins, which constitute the majority of cell proteins, to provide amino acids for gluconeogenesis and energy production during starvation and thus serves physiological functions different from the PKA-mediated increase in the degradation of short-lived proteins.^92,110^ The physiological ability of factors that increase cAMP levels to alter protein turnover and suppress the progression of protein misfolded diseases has not yet been systematically studied.

#### 2.2.4 Increasing Proteasome Activity by Activating cGMP-Dependent Protein Kinase G

Another way to increase proteasome-mediated protein degradation is through cyclic guanosine monophosphate (cGMP) and related protein kinase G (PKG) signaling, similar to cAMP and PKA signaling. cGMP also acts as a second messenger in various cellular processes, including signal transduction and cell growth, but peripheral smooth muscle relaxation is a major medical application.^94,95^ Considerable information and knowledge about the pharmacology and physiology of cAMP and cGMP are available.^91,113−116^ For example, the phosphodiesterase 5 (PDE5) inhibitors sildenafil and tadalafil (Table 1, compounds **6** and **7**) are frequently used to treat pulmonary hypertension and erectile dysfunction, whereas riociguat and the experimental drug BAY41-2272 (Table 1, compounds **8** and **9**), which are stimulators of cGMP synthesis, are also well established.^117,118^ Treating the SH-SY5Y neuroblastoma cell line as well as a number of other cell lines with either specific inhibitors of PDE5 which selectively hydrolyze cGMP, or with a stimulator of soluble guanylate cyclase, such as riociguat and/or BAY41–2272, rapidly increased proteasome activity.^93^ Affinity-purified 26S proteasomes from treated cells hydrolyzed peptides by chymotrypsin-like, trypsin-like, or caspase/peptidyl-glutamyl peptide-like catalytic sites with approximately 2–3-fold increased activity; ATP was hydrolyzed by the 26S proteasome faster; and the ability to degrade a model ubiquitinated substrate was also enhanced. These findings support 19S regulatory particle activation, whose ATPase activity promotes the breakdown of ubiquitin conjugates and the entry of substrates into the 20S particle.^93,94^ However, the essential part of the 26S proteasome that is phosphorylated by PKG and increased catalytic activity has still not been identified. However, the 26S proteasomes activated by cGMP behaved differently than those activated by cAMP. The biochemical assay based on radiolabeled amino acids revealed that increased cGMP levels, like increasing cAMP levels, stimulated the breakdown of short-lived proteins.^94^ Nevertheless, in contrast to cAMP, cGMP significantly enhances the degradation of long-lived proteins without affecting lysosomal proteolysis. A pharmacological increase in cGMP levels, similar to an increase in cAMP levels, enhances 26S proteasome activity and promotes the degradation of aberrant proteins causing neurodegenerative disasters.^94,119^

Treating a mouse model of Charcot-Marie-Tooth disease type 1B (caused by the misfolded mutant myelin protein zero) with the PDE5 inhibitor sildenafil increases proteasome activity in sciatic nerves and reduces the levels of polyubiquitinated proteins, detected by the ubiquitin-dependent proteasome reporter (Ub-G76 V-GFP). Additionally, sildenafil therapy reduced the number of amyelinated axons and increased the thickness of myelin and nerve conduction velocity.^119^ In a cardiomyopathy mouse model caused by the overexpression of mutant αβ-crystalline proteins, treatment with sildenafil led to increased cGMP levels, enhanced proteasome activity, and reduced levels of mutant αβ-Crystallin related to this disease. Thus, increasing cGMP levels may have potential for treating inherited cardiomyopathies.^120,121^ How cGMP levels alter proteasome activity, whether cGMP enhances the breakdown of intracellular proteins in general or in heart tissue only, and whether it influences protein ubiquitination or autophagy were previously unclear. Later, cGMP and PKG were demonstrated to stimulate proteasome activity and cellular proteolysis via the UPS without interfering with autophagy.^94^ Thus, because of their potential to increase proteasome activity and intracellular protein degradation, commonly used pharmacological agents that increase cGMP levels have the potential to combat diseases linked with malfunction in protein degradation and possibly improve the activity of engineered degraders such as PROTACs.

### 2.3 Proteasome Activation by Inhibition of Deubiquitinases

Here, the possibility of increasing proteasome activity through manipulation of an atypical external deubiquitinase is discussed. Two different DUBs with opposing functions are employed for the deubiquitination of the substrate on the proteasome lid. USP14 is a highly effective cysteine protease that reversibly interacts with proteasomes without the need of ATP. It trims ubiquitin chains independent of substrate commitment to proteasome degradation.^122−127^ In contrast, the 19S proteasome lid subunit Rpn11 (also known as PSMD14) recycles polyubiquitin by removing it from protein substrates that are destined for degradation.^122−127^ Rpn11 is a zinc metalloproteinase and an integral proteasome subunit that acts as a positive regulator of degradation, and its activity is linked to ATP-dependent substrate processing.^122−124^ However, the primary regulatory checkpoint of ubiquitin-dependent degradation is the removal of ubiquitin chains from the protein substrates processed by USP14 (Figure 2, C), which reversibly bind to the 26S proteasome and contribute to the editing and rejection of different substrates.^125−129^ USP14 can decrease protein degradation by deubiquitination that results in substrate dissociation from the 26S proteasome. Thus, USP14 inhibition can increase the degradation of ubiquitinated substrates.^130−132^ USP14 is also activated upon proteasome binding, restricting its activity only to this complex. In conclusion, USP14 both regulates and is regulated by the 26S proteasome complex.^131−133^

The inhibition of USP14 by treatment with the small molecule IU1 or its derivative IU1-47 (Table 1, compounds **10** and **11**) enhances the degradation of various neuropathological proteins, including Ataxin-3, Tau, PrP, and TPD-43 in cells.^131,134−137^ A decrease in USP14 activity also confers cytoprotective effects under various neurotoxic conditions.^138−143^ IU1 also dramatically promotes the degradation of oxidized proteins, thereby conferring protection against oxidative damage in HEK293 cells.^31^ In contrast, abolishing the activity of USP14 by siRNA-mediated silencing or the use of small molecule inhibitors leads to the accumulation of polyubiquitinylated proteins and an increase of the apoptotic response in multiple myeloma^145,146^ as well as in different models of solid tumors.^138,144−146^ Increasing proteasome-mediated proteolysis via the inhibition of USP14 also impairs autophagic flux.^142^ These opposing effects of altering USP14 activity might be the result of its diverse cellular functions. Antagonistic effects of USP14 on the proteasome, while initially unexpected, have been observed for many different proteins, including those associated with diseases, for example, in the rescue of a Parkinson’s disease fruit fly model through chemical and genetic inactivation of USP14,^131,134−137,148^ among numerous supporting studies. This finding highlights the potential for the degradation promoting effect of USP14 targeting could be used for therapeutic purposes in proteinopathies, and an opportunity to improve PROTAC activity in the case of proteasome insufficiency and a deeper mechanistic understanding is necessary.

## 3 Challenges

### 3.1 Impact of Proteasome Activation

The proteotoxic burden associated with aging is reduced by increasing proteasome activity in different systems, including humans,^149−151^ in a mouse model, and in contrast, in a naked mole rat (*Heterocephalus glaber*), which is the longest-living known rodent (with a maximum lifetime of approximately 31 years), the proteasomal activity is approximately 1.5-fold greater than that in short-lived mice.^142−155^ Indeed, genetic or pharmacological enhancement of the proteasomal degradation network has been shown to extend lifespan or rescue phenotype in a variety of aging or protein misfolding disease models.^81,93,95,105,119,135,137^ In yeast, in roundworms (*C. elegans*) and fruit flies, or in cell-based systems, genetically enhanced proteasome activity reduces proteotoxic pathologies, delays aging effects, and extends lifespan.^148,156−169^ This observation is further supported by data from human healthy centenarians’ fibroblasts, whose proteasome activity is greater than in fibroblasts from adults of different ages.^150^ In short-lived African turquoise killifish (*Nothobranchius furzeri*), a decline in proteasome activity is an early event during brain aging and is sufficient to induce aging-related proteomic changes and loss of stoichiometry *in vivo*; moreover, an early life decline in proteasome levels is a serious risk factor for premature mortality.^170^ Importantly, studies using short-term exposure of cells to low-molecular-weight proteasome activators have not shown inherent toxicity; however, only one study has described some long-term effects.^168,170^ After 12 weeks of continuous treatment of human embryonic fibroblasts with the proteasome activator oleuropein, which has an unknown mechanism of action, the number of oxidized proteins was reduced.^163^ Genetic activation of proteasome activity is achieved by stable overexpression of the β5 catalytic subunits (responsible for chymotrypsin-like activity) in primary human fibroblast lines, resulting in elevated levels of other β-type subunits and enhanced of all three 20S proteasome enzymatic activities. Importantly, β5 overexpression allows fibroblasts to significantly expand their lifespan. β5 overexpression also has a similar effect on lifespan in a fruit fly model.^171,172^ Similarly, the recovery of normal proteasome catalytic subunit levels through lentiviral transduction alleviates multiple aspects of aging in human dermal fibroblasts from aged donors.^173^ Transgenic mice with neuronal-specific overexpression of the proteasome β5 subunit were crossed with Alzheimer’s disease model mice. The final hybrids showed reduced mortality and cognitive deficits.^174^ However, in the case of USP14 deubiquitinase inhibition, the effects on cell viability differ depending on the cell type.^135−146^

Several other studies have aimed to stimulate 26S activity and increase proteasome complex levels; however, to date, no studies have evaluated the intrinsic activation of the 20S proteasome itself. Recently, this aspect has been described in the roundworm *C. elegans*, which endogenously expresses a hyperactive, open gate proteasome. These mutant worms were altered in the germline to result in a viable strain with enhanced proteasome activity, displaying increased peptide, unstructured protein, and ubiquitin-dependent degradation activities. Moreover, this strain exhibited a markedly increased lifespan and substantial resistance to proteotoxic and oxidative stress. These results demonstrate that the introduction of a hyperactive proteasome into a multicellular organism is achievable and reveal that targeting the proteasome gating mechanism as a promising approach for future age-associated disease research.^163^

The inner space of the substrate translocation channel of the proteasome core particle is limited by the convergent N-termini of the α-subunits. In mammalian cells, α3 subunits with deleted N-terminal tails were shown to form intact but hyperactive proteasomes based on tracking the hydrolysis of fluorogenic peptide substrates and the degradation of polyubiquitin chain labeled proteins.^175^ Cells with constitutively activated proteasomes exhibit significantly increased degradation of numerous proteasome substrates and are more resistant to oxidative stress. Quantitative proteomics demonstrated that approximately two hundred proteins whose level was reduced in proteasome open-gate mutant cells. Tau and other potentially harmful proteins exhibit reduced accumulation and aggregation.^175^ These findings indicate that stimulating proteasome activity has diverse effects on the clearance of different proteins and that there are no significant toxic effects at the cellular or organismal level.^163,175^ However, it has also been shown that a hyperactivated proteasome reduces the fecundity of mutant worm strain.^163^

## 4 Conclusion

In general, proteasome flux is not fixed but rather flexible due to the ability of cells to adapt to the needs of different physiological conditions, which in turn affects overall proteolytic capacity. The capacity of cells to degrade proteins via the 26S proteasome can be promoted by two distinct mechanisms: an increase in the proteasome content or an alteration of its catalytic activity. The first way to restore proteostasis is through the activation of NRF1, a transcription factor that promotes the coordinated expression of all proteasome component genes to prevent proteasome malfunction and, in some cases, increases its activity. The other ways to activate proteasome-mediated proteolysis include cAMP and cGMP, which are second messengers, as well as by suppressing p38-MAPK related signaling. The pharmacological targeting of these signaling pathways can cause rapid activation of proteasomes and increased protein degradation in different cell types or tissues and rescue phenotypes in various models of human protein conformation diseases. Inhibition of the majority of DUBs prevents ubiquitin-labeled proteins from escaping their fate. Blocking the proteasome external deubiquitinase USP14 can promote the degradation of ubiquitinated substrates and help to maintain proteostasis during multiple stress events. Although there are not currently approved therapies based on deubiquitinase inhibitors, this topic is an emerging field with enormous potential. Herein, I further investigated the potential of pharmacological activation of the proteasome to reduce the proteotoxicity linked with many pathological processes as well as its potential applicability for improving the efficacy of the PROTAC strategy, mainly for treating disorders characterized by proteasome malfunction and for drug repurposing. Unfortunately, this field is still in its early stages, and positive outcomes and clinical applications still do not exist.

## Abbreviations

ATP: Adenosine triphosphate
ACs: Adenylate cyclases
ADH: Antidiuretic hormone
ARE: Antioxidant response element
ASK1: Apoptosis signal-regulating kinase 1
βTrCP1: Betatransducin repeat-containing E3 ubiquitin protein ligase
BDR4: Bromodomain containing protein 4
CaMKII: Calcium/calmodulin-dependent protein kinase II
c-IAP1: Cellular inhibitor of apoptosis protein 1
CRBN: Cereblon
PKG: cGMP-dependent protein kinase
CRL: Cullin-RING ligase
cAMP: Cyclic adenosine monophosphate
cGMP: Cyclic guanosine monophosphate
DCAF15: DDB1 and CUL4 associated factor 15
DCAF16: DDB1 and CUL4 associated factor 16
DUB: Deubiquitinase
DDI2: DNA damage inducible 1 homologue 2
RNF114: E3 ubiquitin-protein ligase RNF114
FDA: U.S. Food & Drug Administration
FOXO: FOX (forkhead box) protein
GSPT1: G1-to-S phase transition 1
HTT: Huntingtin
IKZF1: Ikaros family zinc finger protein 1
IKZF3: Ikaros family zinc finger protein 3
IMiD: Immunomodulatory drug
PrP: Major prion protein
MKK6: MAP kinase kinase 6
LC3: Microtubule associated protein 1 light chain 3 beta
MK2: Mitogenactivated protein kinase (MAPK)-activated protein kinase 2
Mdm2: Mouse double minute 2 homologue
MAF: Musculoaponeurotic fibrosarcoma protein
NGLY1: N-glycanase 1
NFE2L1: Nuclear factor erythroid 2-related factor 1
NFE2L2: Nuclear factor erythroid 2-related factor 2
p38-MAPK: p38 mitogen-activated protein kinase
PDE5: Phosphodiesterase 5
PDE4: Phosphodiesterase-4
PSME1: Proteasome activator subunit 1
PKA: Protein kinase A
PKG: Protein kinase G
POI: Protein of interest
SKN-1A: Protein skinhead-1A
PROTAC: Proteolysis targeting chimera
SALL4: Sal-like protein 4
TPD-43: TAR DNA-binding protein 43
UPS: Ubiquitin–proteasome system
pVHL: von Hippel–Lindau protein
Ub: Ubiquitin
USP14: Ubiquitin-specific protease 14
(VCP)/p97: Valosin-containing protein
ZFAND5: Zinc finger A20 domain-containing protein 2
